# Local brain-state dependency of effective connectivity: a pilot TMS–EEG study

**DOI:** 10.12688/openreseurope.14634.2

**Published:** 2022-07-11

**Authors:** Ida Granö, Tuomas P. Mutanen, Aino Tervo, Jaakko O. Nieminen, Victor H. Souza, Matteo Fecchio, Mario Rosanova, Pantelis Lioumis, Risto J. Ilmoniemi

**Affiliations:** 1 Department of Neuroscience and Biomedical Engineering, Aalto University School of Science, Espoo, Finland; 2 BioMag Laboratory, HUS Medical Imaging Center, University of Helsinki and Helsinki University Hospital, Helsinki, Finland; 3 School of Physiotherapy, Federal University of Juiz de Fora, Juiz de Fora, MG, Brazil; 4 Department of Biomedical and Clinical Sciences “L. Sacco”, University of Milan, Milan, Italy; 5 Center for Neurotechnology and Neurorecovery, Department of Neurology, Massachusetts General Hospital, Boston, MA, USA

**Keywords:** Transcranial magnetic stimulation; electroencephalography; brain state; effective connectivity

## Abstract

**Background: **Spontaneous cortical oscillations have been shown to modulate cortical responses to transcranial magnetic stimulation (TMS). However, whether these oscillations influence cortical effective connectivity is largely unknown. We conducted a pilot study to set the basis for addressing how spontaneous oscillations affect cortical effective connectivity measured through TMS-evoked potentials (TEPs).

**Methods: **We applied TMS to the left primary motor cortex and right pre-supplementary motor area of three subjects while recording EEG. We classified trials off-line into positive- and negative-phase classes according to the mu and beta rhythms. We calculated differences in the global mean-field amplitude (GMFA) and compared the cortical spreading of the TMS-evoked activity between the two classes.

**Results: **Phase affected the GMFA in four out of 12 datasets (3 subjects × 2 stimulation sites × 2 frequency bands). Two of the observed significant intervals were before 50 ms, two between 50 and 100 ms, and one after 100 ms post-stimulus. Source estimates showed complex spatial differences between the classes in the cortical spreading of the TMS-evoked activity.

**Conclusions: **TMS-evoked effective connectivity seems to depend on the phase of local cortical oscillations at the stimulated site. This work paves the way to design future closed-loop stimulation paradigms.

## Introduction

The state of the brain affects the efficacy of transcranial magnetic stimulation (TMS; ^1– 10^) in eliciting cortical responses, such as those observed by means of TMS combined with electroencephalography (TMS–EEG). For instance, TMS–EEG can reveal effective connectivity patterns depending on sleep stage or deep sedation ^1, 4^. Noting that EEG signals provide a measure of brain state (projection of post-synaptic currents; ^11, 12^), we focus on the phase of oscillatory signals that reflect the local brain state and its impact on effective connectivity patterns.

Moreover, pre-stimulus oscillations can modulate TMS-evoked potentials (TEPs) ^3, 9, 13, 14^, and if not accounted for, within-subject variability may mask meaningful changes in reactivity and measures of connectivity ^15^. To address these challenges, brain-state-dependent and closed-loop stimulation paradigms are being developed ^16– 26^. To benefit fully from these novel techniques, we need to understand the basic mechanisms through which oscillations modulate cortical effective connectivity.

Both mu and beta rhythms (8–13 Hz, 13–30 Hz, respectively) in the frontal lobe can modulate TMS cortical and corticospinal responses ^9, 13, 27– 31^. In this preliminary work, we investigate the role of the phase of these two rhythms in effective connectivity when stimulating the left primary motor cortex (M1) and the right pre-supplementary motor areas (pre-SMA). As an indicator of effective connectivity, we investigate TMS-induced signal propagation, *i.e*., the spatio-spectral patterns of TMS-evoked activity spreading across the cortex.

## Methods

### Data acquisition

Three healthy right-handed volunteer subjects (S1, female, 28 years old; S2, male, 41; S3, male, 43) were recruited. The Coordinating Ethics Committee of Helsinki University Hospital approved the study, and all subjects signed a written informed consent. During the experiment, the subject sat in a comfortable chair, fixating on a black cross 3 m away. To prevent the perception of the click sound produced by the TMS pulse, the subject wore earmuffs ^4, 32– 34^ over in-ear earphones that continuously played white noise combined with random bursts of recorded TMS click sounds ^35^.

Biphasic TMS pulses were delivered through a figure-of-eight coil (70-mm radius; Cooled Coil, Nexstim Plc, Finland) connected to a Nexstim NBS 4.3 eXimia stimulator. Coil positioning was guided by neuronavigation software (Nexstim) based on the individual’s T1-weighted magnetic resonance images (MRI). EEG signals were recorded with 60 Ag/AgCl-sintered electrodes and a TMS-compatible amplifier ( 36; eXimia EEG, Nexstim), bandpass-filtered at 0.1–350 Hz, and sampled at 1450 Hz. The scalp under the electrodes was scraped with conductive abrasive paste (OneStep AbrasivPlus, H + H Medical Devices, Germany) before the electrodes were filled with conductive gel (Electro-Gel, ECI, Netherlands). Each electrode’s impedance was kept below 5 kΩ. The reference and ground electrodes were placed on the right mastoid and zygomatic bone, respectively. Motor-evoked potentials (MEPs) were recorded with a Nexstim electromyography (EMG) system. The EMG electrodes were fixed in a belly–tendon montage on the right *abductor pollicis brevis* (APB) muscle. Before the TMS–EEG experiment, we determined for each subject the optimal coil location and orientation producing the largest MEP with a fixed suprathreshold intensity ^37, 38^. At the optimal location, we estimated the resting motor threshold (RMT) as the intensity producing MEPs larger than 50 µV in 5 out of 10 times ^39^.

Single-pulse TMS was applied to the left M1 at the cortical representation site of APB and the right pre-SMA. For M1, we used an initial TMS intensity of 90% of RMT. We rotated and moved the coil to minimize any remaining peripheral responses (MEPs) and scalp muscle activations in the EEG ^40^. Additionally, we used a dedicated real-time EEG readout ^41^ to fine-tune the stimulation intensity to obtain an early (<50 ms) response nearby the stimulated target with a peak-to-peak amplitude of 6–10 μV on average reference after averaging 20 trials. If MEPs were still present, we relocated the coil more medially within the motor knob. This resulted in stimulation intensities of 60 V/m for S1, 55 V/m for S2, and 90 V/m for S3 ^42^.

The pre-SMA rough stimulation area was identified by individual anatomical landmarks as described earlier ^34, 43^. The final stimulation parameters were adjusted based on the output of a dedicated real-time EEG readout, a procedure followed as well for M1 ^41^. The final stimulation intensities at pre-SMA for subjects S1, S2, and S3 were 100, 80, and 125 V/m, respectively. The stimuli were given at random interstimulus intervals of 2–2.3 s; a block of 250 pulses was delivered to each target per subject. The sample-and-hold electronics of the EEG device ^36^, and the iterative process to adjust the coil location and orientation resulted in minimal TMS-related artifacts in the EEG recording for both stimulation locations.

### Pre-processing

Data were pre-processed with custom-made MATLAB 2019a scripts ^44^ based on the EEGLAB toolbox ^45^. The signals were first filtered at 1–45 Hz with a third-order zero-phase-shift Butterworth bandpass filter. Then, epochs were extracted with a time window of −1 to 1 s relative to the TMS pulse. After visual inspection, we removed trials heavily contaminated by eye blinks or scalp-muscle activations. Then, data were re-referenced to the average potential, and the baseline was corrected by subtracting the baseline average (−1000…−2 ms). Next, independent component analysis (ICA) separated the data into predominantly artefactual and neuronal components. These components were visually inspected for every trial. Trials with highly distorted components were rejected, and then ICA was recomputed on the remaining data (number of remaining trials, after both trial-rejection steps: (mean±sd 233±11, range 218–244). Independent components generated by eye blinks, eye movements, continuous muscle artifacts unrelated to TMS timing, and electrode-movement artifacts were removed (mean±sd: 12±2 components were removed per dataset).

### Phase evaluation

The trials were split semi-manually into positive- and negative-phase classes, separately for mu and beta bands, based on the pre-stimulus phase in each trial. First, the signals were bandpass filtered with a 4th-order zero-phase-shift Butterworth filter in the frequency band of interest. Then, a Hilbert transform was applied to determine the instantaneous phase at the time of the TMS pulse. Trials with a maximum deviation of 30° from the peaks were set into positive-phase or negative-phase classes, respectively. To correct for cases where the narrow-band signal did not correspond well to the broadband one, we manually inspected the choices made by the algorithm and corrected them in cases of clear misclassification. For this, both raw and the bandpass-filtered signals at the frequencies of interest were displayed from channel C3 (when stimulating M1) or F2 (when stimulating pre-SMA), together with the decision made by the algorithm. A trial was reclassified as positive- or negative-phase if the phase difference between the instantaneous phase at the TMS onset and the positive or negative peak, respectively, was less than 40°, and the unfiltered signal was qualitatively similar in waveform to the filtered one. Trials were excluded from further analysis if the signals greatly differed or TMS occurred at some other phase. We obtained for the analysis 72.6±20.5 (mean±sd) trials in each class and a total of 12 datasets (2 stimulus locations × 3 subjects × 2 frequency bands).

### Correction of background oscillatory activity

Typically, the TMS-evoked responses are estimated as the mean across trials that have been delivered at randomized time intervals. The rationale is that, in this case, any background oscillations that are not time-locked to the stimulus are attenuated by the averaging process. However, in trials classified according to the pre-stimulus phase, such background oscillations are consistent across trials and are consequently present in the averaged signal. This effect, if not adequately addressed, may lead to incorrect interpretations. We removed the phase classification effect by extracting the pre-stimulus time period (−1000…0 ms) of each trial, sorting these non-stimulated trials according to phase at −500 ms, and subtracting their mean from the stimulated trials ^14, 46, 47^. The stimulated trials were cut to a length of −500…500 ms when applying the correction to match the non-stimulated trials’ length.

### Source analysis

For each dataset, the global mean-field amplitude (GMFA ^48, 49^) was computed. To compare the two classes, we calculated the absolute difference in their GMFAs (|GMFA _positive phase_ – GMFA _negative phase_|), and set a threshold based on 1000 random reassignments of the trials into new pseudoclasses. For each permutation, the maximum absolute difference between the pseudoclasses was calculated and stored. This procedure controls the within-dataset false discovery rate ^50^. To keep the total false discovery rate below 0.05, we applied the Benjamini–Hochberg procedure ^51^ to set the threshold at the corresponding percentile of the permutation distribution for each dataset with (1 – *r* * 0.05/12), where *r* is the rank of the dataset, and 12 is the total number of datasets. The rank was determined by the maximum difference in GMFA between the classes with respect to the permuted distributions. For time intervals where the differences in the GMFAs between the positive- and negative-phase classes in the post-TMS time period (0…300 ms) exceeded this threshold, we conducted source estimation. We averaged the mean EEG responses in these time intervals for both classes separately, which were then utilized for Tikhonov-regularized minimum-norm estimates (MNE) ^52^. The obtained MNE maps were thresholded for visualization to show only the cortical area corresponding to at least 60% of the maximum MNE amplitude.

For source estimation, we calculated the lead fields that describe the sensitivity profiles of different EEG channels to neuronal activity in all the plausible cortical locations. First, the scalp, skull, and white-matter surfaces were extracted from the MRIs using the *headreco* ^53– 55^ function of the SimNIBS software ^56^. The surface meshes were imported to MATLAB, decimated to ~10,000 nodes, and cleaned from surface artifacts using the iso2mesh package ^57^. The lead-field matrices were calculated with the boundary element method assuming conductivity values of 0.33, 0.0033 and 0.33 S/m for the intracranial cavity, skull and scalp, respectively ^58^. Focal post-synaptic currents were modeled as current dipoles oriented normal to the white matter surface. For obtaining the cortical activity estimates, the Tikhonov-regularized MNE was used for projecting the TEPs to the source space ^52^ with a regularization parameter of 0.1.

## Results

### TEPs and GMFAs

**Figure 1.**
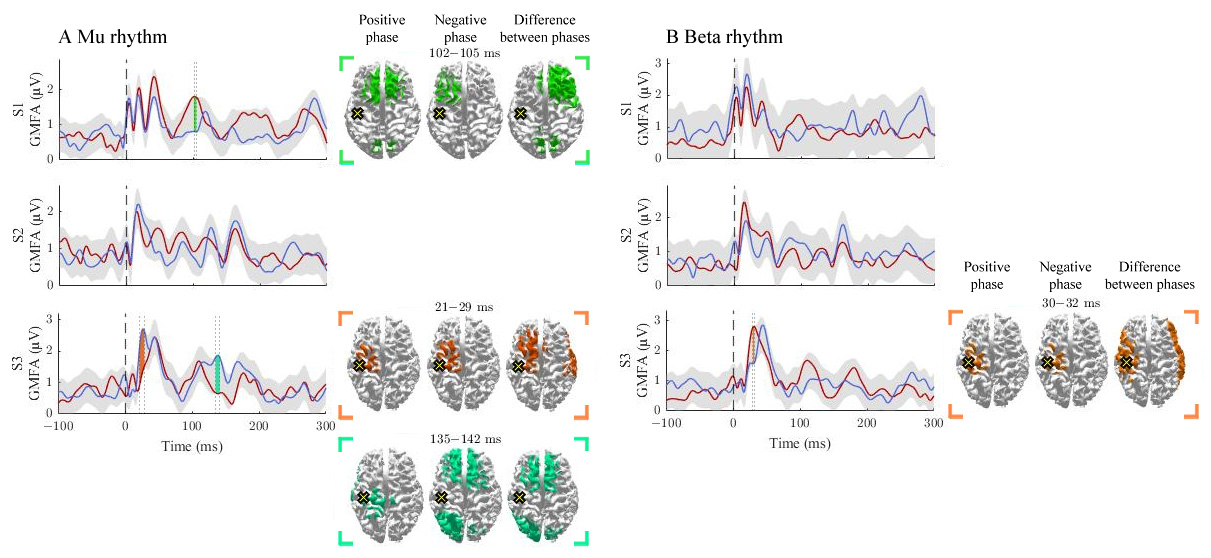
The effect of the positive and negative phases on TEPs when stimulating M1. The **A** and **B** panels summarize the effects of mu and beta rhythms, respectively. The curves show the global mean-field amplitudes (GMFA) of the positive-phase (red) and negative-phase (blue) conditions. The cortical maps illustrate the source estimates for the significant differences between the phase conditions. The shaded areas indicate the average GMFA over the two conditions ± the threshold for meaningful changes. Time intervals that exceed the threshold are marked with different colors. For each time interval, the corresponding time-averaged source estimates are shown on the right in the same color. For each time interval, only sources stronger than 60% of the maximum amplitude are shown. The dark dashed vertical line indicates the time of the TMS pulse. The cross marks the stimulation site.

**Figure 2.**
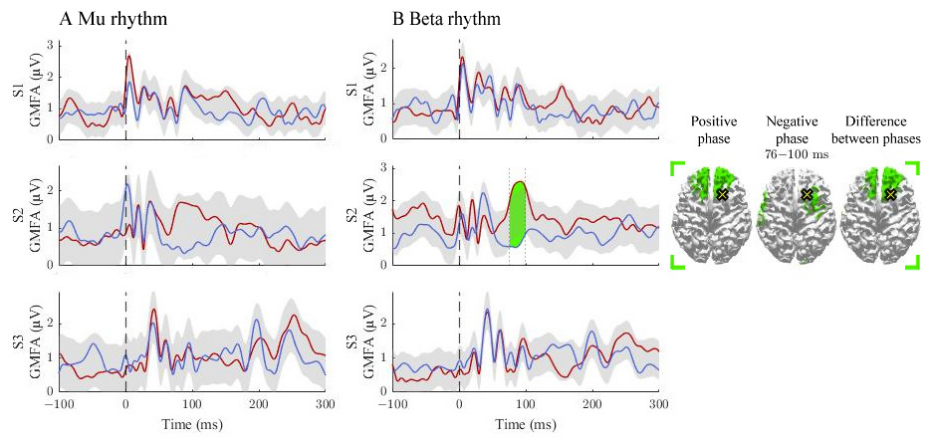
The effect of the positive and negative phases on TEPs when stimulating pre-SMA. The **A** and **B** panels summarize the effects of mu and beta rhythms, respectively. The curves show the global mean-field amplitudes (GMFA) of the positive-phase (red) and negative-phase (blue) conditions, whereas the cortical maps illustrate the source estimates for the significant differences between the phase conditions. The shaded areas indicate the average GMFA over the two conditions ± the threshold for meaningful changes. The time interval which exceeds the set threshold is marked with color. For the time interval, the corresponding time-averaged source estimates are shown on the right in the same color. Only sources stronger than 60% of the maximum amplitude are shown. The dark dashed vertical line indicates the time of the TMS pulse. The cross marks the stimulation site.

We observed differences in GMFAs between the positive- and negative-phase classes in 4/12 comparisons that exceeded the threshold level. Two of the observed significant intervals were before 50 ms, two between 50 and 100 ms, and one after 100 ms post-stimulus ( Figure 1 and Figure 2). Source estimates showed the most abundant differences close to the stimulation site. We observed large inter-individual variability in the spatial and temporal characteristics of the phase effects.

### Signal propagation after M1 stimulation

The activation patterns and differences between the negative- and positive-phase classes are illustrated in Figure 1. The mu rhythm modulated responses in S1 and S3. In S1, the positive-phase condition elicited larger responses than the negative-phase condition at 102–105 ms post-stimulus. In S3, the negative-phase condition produced larger GMFAs at 21–19 and 135–142 ms post-stimulus. The beta rhythm modulated responses only in S3 at 30–32 ms post-stimulus, at the stimulation site and in the lateral right hemisphere, where the positive-phase condition produced larger GMFAs than the negative one. 

The beta rhythm modulated responses only in S3 at 30–32 ms post-stimulus, at the stimulation site and in the lateral right hemisphere, where the positive-phase condition produced larger GMFAs than the negative one.

### Signal propagation after pre-SMA stimulation

The activation patterns and differences between the classes are illustrated in Figure 2. For the mu rhythm, no supra-treshold time-intervals were found. The beta rhythm modulated responses in S2 at 76–100 ms post-stimulus, where the positive-phase condition elicited stronger responses than the negative one. The source estimates revealed differences close to the stimulation site.

## Discussion

We found that the phase of spontaneous cortical oscillations at the TMS pulse instant seems to affect the post-stimulus effective connectivity pattern. It is proposed that the state of the post-synaptic neural population modulates the efficacy of the synaptic transmission ^59^. Such mechanisms can play a role in multiple places in the signaling cascade, determining where and when the responses differ from each other. We observed high variability between subjects, which could be credited to, *e.g*., differences in the cortical folding, inter-individual differences in stimulated circuits, and inter-individual cortical connections.

To highlight meaningful changes due to the phase of ongoing EEG oscillations on TEPs, we analyzed differences in GMFA that are unlikely to reflect purely changes in the background activity. In this preliminary study, we observed supratreshold differences in 4 out of 12 datasets already with this small number of trials. More data would likely show more subtle phase effects not distinguishable with this trial number. Our *post hoc* power analysis ^60^ indicated that assuming a short-lived (20 ms) 1-µV difference in GMFA, we would need over 100 trials in each phase class to show this difference statistically with 80% power. It is also important to note that three subjects is a relatively small sample size and our interpretations may not be generalizable for a larger population. Nonetheless, three subjects are sufficient for demonstrating the methodology and at a single subject level a possible effect of ongoing oscillations on the effective connectivity. Thus, in future studies, we need to collect a higher number of trials per phase in a larger group of study participants to consolidate our observations.

Other pre-stimulus indices than the phase have also been shown to modulate effective connectivity in the human corticocortical circuits ^30, 61– 64^. These same factors could also play a role in corticocortical effective connectivity. For example, high pre-stimulus mu power has been shown to reduce MEP amplitudes ^30, 63^, although more research is still needed ^65^. Power has also been suggested to interact with the phase, resulting in power-dependent phase modulation ^29^. Therefore, further control of the power in phase-dependent stimulation will be important in future works.

## Conclusions

Our results suggest that TMS-induced effective connectivity is dependent on the pre-stimulus phase of the local oscillations. Our findings open new avenues for further research and support the progress of brain-state-dependent and closed-loop stimulation paradigms.

## Institutional Review Board Statement

**Ethics and Consent:** The study was conducted according to the guidelines of the Declaration of Helsinki, and approved by The Coordinating Ethics Committee of Helsinki University Hospital (protocol code: HUS/1198/2016, date of approval: 21.7.2017). Written informed consent was obtained from all subjects involved in the study.

## Data availability

The data presented in this study are available upon reasonable request from the corresponding author as long as the confidentiality requirements are strictly followed. We are not allowed to make physiological or anatomical data publicly available according to our ethical permission statement.
