# Supplementary material for: Local brain-state dependency of effective connectivity: a pilot TMS–EEG study
Source: Open Res Eur. 2022 Jul 11;2:45. Originally published 2022 Apr 7. [Version 2] doi: 10.12688/openreseurope.14634.2 (PMC7613446; doi:10.12688/openreseurope.14634.2)
Supplement: Peer review history

# Peer review: Reviewer response for version 2

**DOI:** 10.21956/openreseurope.16165.r29686

**Published:** 2022-08-04

**Authors:** Keiichi Kitajo

**Affiliations:** 1 Division of Neural Dynamics, Department of System Neuroscience, National Institute for Physiological Sciences, National Institutes of Natural Sciences, Okazaki, Japan

The authors have addressed all of my concerns. I think that the manuscript is acceptable.

Is the study design appropriate and does the work have academic merit?

Yes

Is the work clearly and accurately presented and does it cite the current literature?

Yes

If applicable, is the statistical analysis and its interpretation appropriate?

No

Are all the source data underlying the results available to ensure full reproducibility?

Partly

Are the conclusions drawn adequately supported by the results?

Partly

Are sufficient details of methods and analysis provided to allow replication by others?

Partly

Reviewer Expertise:

Nonlinear neural dynamics, Computational Neuroscience, Cognitive Neuroscience

I confirm that I have read this submission and believe that I have an appropriate level of expertise to confirm that it is of an acceptable scientific standard.

# Peer review: Reviewer response for version 2

**DOI:** 10.21956/openreseurope.16165.r29685

**Published:** 2022-08-02

**Authors:** Florinda Ferreri

**Affiliations:** 1 Department of Neuroscience, University of Padua, Padova, Italy

I have read the new version of the paper. It can be indexed as is.

Is the study design appropriate and does the work have academic merit?

Partly

Is the work clearly and accurately presented and does it cite the current literature?

Partly

If applicable, is the statistical analysis and its interpretation appropriate?

Yes

Are all the source data underlying the results available to ensure full reproducibility?

Partly

Are the conclusions drawn adequately supported by the results?

Partly

Are sufficient details of methods and analysis provided to allow replication by others?

Yes

Reviewer Expertise:

TMS, TMS-EEG

I confirm that I have read this submission and believe that I have an appropriate level of expertise to confirm that it is of an acceptable scientific standard.

# Peer review: Reviewer response for version 1

**DOI:** 10.21956/openreseurope.15803.r29330

**Published:** 2022-06-21

**Authors:** Florinda Ferreri

**Affiliations:** 1 Department of Neuroscience, University of Padua, Padova, Italy

In the paper “Local brain-state dependency of effective connectivity: a pilot TMS–EEG study” the Authors aimed to conduct a pilot study to set the basis for addressing how spontaneous oscillations affect cortical effective connectivity as measured through TMS-evoked potentials (TEPs).” They applied TMS to the left primary motor cortex and right pre-supplementary motor area of three subjects while recording EEG. They conclude that TMS-evoked effective connectivity appears to depend on the phase of local cortical oscillations at the stimulated site.

 The paper is interesting, but 3 subjects are not enough even for a pilot study. I have some further comments:

- It is not so clear the phrase “we rotated and moved the coil to minimize both peripheral responses (MEPs) and scalp muscle activations”. At the 90% of RMT no MEPs are expected.
- After stimulation of M1, TEPs have been clearly described: which component is expected to have a peak-to-peak amplitude of 6–10 μV after averaging 20 trials?  
- In TMS-EEG experiments Independent component analysis (ICA) can cancel genuine brain responses. The Authors should comment further on this point.  

Is the study design appropriate and does the work have academic merit?

Partly

Is the work clearly and accurately presented and does it cite the current literature?

Partly

If applicable, is the statistical analysis and its interpretation appropriate?

Yes

Are all the source data underlying the results available to ensure full reproducibility?

Partly

Are the conclusions drawn adequately supported by the results?

Partly

Are sufficient details of methods and analysis provided to allow replication by others?

Yes

Reviewer Expertise:

TMS, TMS-EEG

I confirm that I have read this submission and believe that I have an appropriate level of expertise to state that I do not consider it to be of an acceptable scientific standard, for reasons outlined above.

# Response

**Published:** 2022-07-05

**Authors:** Pantelis Lioumis

**Affiliations:** Aalto University School of Science, Finland

*In the paper “Local brain-state dependency of effective connectivity: a pilot TMS–EEG study” the Authors aimed to conduct a pilot study to set the basis for addressing how spontaneous oscillations affect cortical effective connectivity as measured through TMS-evoked potentials (TEPs).” They applied TMS to the left primary motor cortex and right pre-supplementary motor area of three subjects while recording EEG. They conclude that TMS-evoked effective connectivity appears to depend on the phase of local cortical oscillations at the stimulated site*.

*The paper is interesting, but 3 subjects are not enough even for a pilot study. I have some further comments:*

**Answer:** We thank the reviewer for the interest in our paper. Regarding the number of subjects; yes, 3 is a small number. However, many discoveries have been made with 3 or even fewer subjects. In this case, we wanted to demonstrate a phenomenon and propose a methodology rather than to obtain statistics about how common this type of finding is on a population level. We have now addressed this in the Discussion section: “It is also important to note that three subjects is a relatively small sample size and our interpretations may not be generalizable for a larger population. Nonetheless, three subjects are sufficient for demonstrating the methodology and at a single subject level a possible effect of ongoing oscillations on the effective connectivity. Thus, in future studies, we need to collect a higher number of trials per phase in a larger group of study participants to consolidate our observations.”

*It is not so clear the phrase “we rotated and moved the coil to minimize both peripheral responses (MEPs) and scalp muscle activations”. At the 90% of RMT no MEPs are expected.*

**Answer:** MEPs can be present even with 90% of RMT; they are simply appearing more seldomly and with smaller amplitudes. This is what we observed in some of the subjects, and this is why we applied this procedure to minimize any remaining MEPs as well as scalp muscle activations. We have clarified this in the Data acquisition section: “We rotated and moved the coil to minimize any remaining peripheral responses (MEPs) and scalp muscle activations in the EEG.”

*After stimulation of M1, TEPs have been clearly described: which component is expected to have a peak-to-peak amplitude of 6–10 μV after averaging 20 trials?*

**Answer:** This aim of 6–10 µV peak-to-peak amplitudes refers to the earliest distinguishable components. For M1, that would be the N15–P30 peak-to-peak amplitudes in most subjects. We defined this criterion as peak-to-peak amplitudes before 50 ms for it to be applicable also to other stimulation sites, where peak latencies can vary, such as in pre-SMA in our case (usually around P20 and N40).

*In TMS-EEG experiments Independent component analysis (ICA) can cancel genuine brain responses. The Authors should comment further on this point.*

**Answer:** Yes, this is completely correct. Independent component analysis (ICA) attempts to separate events in the data that are independent in time. Brain signals can especially mix with artefacts time-locked to the TMS pulse in ICA, as these do not meet the requirement of independence (Metsomaa *et al.*, 2014). Therefore, we only used ICA to remove events that are independent to the timing of the TMS pulse, such as ocular artefacts and continuous scalp muscle activations. ICA, when used with caution, can be a useful tool for removing certain types of artefacts, and is widely utilized in TMS–EEG analysis. It is also important to note that, when running the experiments, we applied procedures to minimize artefacts such as scalp muscle activations by fine tuning the stimulation parameters, as explained in the Data acquisition section. This way we guaranteed the safe use of ICA as we did not need to remove such artefacts with it. Metsomaa, J., Sarvas, J., Ilmoniemi, R.J., 2014. Multi-trial evoked EEG and independent component analysis. J. Neurosci. Methods 228, 15-26. doi:10.1016/j.jneumeth.2014.02.019

# Peer review: Reviewer response for version 1

**DOI:** 10.21956/openreseurope.15803.r28993

**Published:** 2022-04-25

**Authors:** Keiichi Kitajo

**Affiliations:** 1 Division of Neural Dynamics, Department of System Neuroscience, National Institute for Physiological Sciences, National Institutes of Natural Sciences, Okazaki, Japan

The authors report the effects of the instantaneous phase of EEG oscillations on the effective connectivity as assessed by the global cortical spread of TMS-evoked responses targeting two local areas, right M1 or pre-SMA. The research question is sound. Although this study uses cutting-edge techniques to measure and assess TMS-evoked responses, it is hard to conclude things using data from three participants. Still, it will be beneficial for readers to read this manuscript who are interested in the methodological aspects of TMS-EEG recordings and phase-dependent neural dynamics. I, therefore, suggest the authors provide more technical details of the study.

 How did the authors determine the 90% RMT?

 On page 3, For M1, we applied an initial TMS intensity of 90% of RMT. We rotated and moved the coil to minimize both peripheral responses (MEPs) and scalp muscle activations.

 I don’t quite get the exact procedure to determine 90% of RMT. I suppose that the regular procedure is as the following. First, the coil orientation and location should be optimized to get the most prominent MEP at a TMS intensity higher than the RMT. Next, the RMT should be determined by some adaptive procedure reducing the TMS intensity. Finally, the intensity of TMS should be reduced to 90% of RMT, keeping the orientation and location of the coil. Is the authors' procedure different from this?

 How was the TMS intensity for pre-SMA optimized for each individual?

 I think that one of the challenges in TMS-EEG studies is artifact reduction. However, I don’t see details on the online and offline methodology of artifact reduction except for auditory artifact reduction using in-ear earphones and earmuffs. Were the lead wires rearranged relative to the coil orientation to reduce TMS-induced electromagnetic artifacts?

 How were the TMS-evoked artifacts, such as the decay artifact, removed in the offline analysis? Did the EEG amplifier system have any online artifact reduction protocol? Please explain these details more explicitly.

 On page 4, I understand that the authors conducted trial-wise permutation tests to analyze the GMFA differences between two target phase conditions within each subject. And the authors corrected the results by the number of multiple comparisons, which the authors think is 12, i.e., the number of datasets. However, if the authors conduct the permutation test at every time point, the number of comparisons will be much larger considering the number of time points. What is the rationale for ignoring the number of time points? Another idea is to go for the cluster-based permutation test (Maris *et al.* 2007 ^1^) considering the cluster. I am not sure if there is a single-subject version of the statistical test, though.

 Figures 1 and 2. The titles for the figures look exactly the same. I guess the authors forgot to put M1 and pre-SMA.

Is the study design appropriate and does the work have academic merit?

Yes

Is the work clearly and accurately presented and does it cite the current literature?

Yes

If applicable, is the statistical analysis and its interpretation appropriate?

No

Are all the source data underlying the results available to ensure full reproducibility?

Partly

Are the conclusions drawn adequately supported by the results?

Partly

Are sufficient details of methods and analysis provided to allow replication by others?

Partly

Reviewer Expertise:

Nonlinear neural dynamics, Computational Neuroscience, Cognitive Neuroscience

I confirm that I have read this submission and believe that I have an appropriate level of expertise to state that I do not consider it to be of an acceptable scientific standard, for reasons outlined above.

## References

[1] : Nonparametric statistical testing of EEG- and MEG-data. *J Neurosci Methods* .2007;164(1) : 10.1016/j.jneumeth.2007.03.024 177-90 10.1016/j.jneumeth.2007.03.024 17517438

# Response

**Published:** 2022-04-30

**Authors:** Pantelis Lioumis

**Affiliations:** Aalto University School of Science, Finland

The authors report the effects of the instantaneous phase of EEG oscillations on the effective connectivity as assessed by the global cortical spread of TMS-evoked responses targeting two local areas, right M1 or pre-SMA. The research question is sound. Although this study uses cutting-edge techniques to measure and assess TMS-evoked responses, it is hard to conclude things using data from three participants. Still, it will be beneficial for readers to read this manuscript who are interested in the methodological aspects of TMS-EEG recordings and phase-dependent neural dynamics. I, therefore, suggest the authors provide more technical details of the study.

**Answer:** We thank the reviewer for the positive comments and for an encouraging review. Below, we answer each point separately. How did the authors determine the 90% RMT? We answer this question below the next comment.

 On page 3, For M1, we applied an initial TMS intensity of 90% of RMT. We rotated and moved the coil to minimize both peripheral responses (MEPs) and scalp muscle activations.

 I don’t quite get the exact procedure to determine 90% of RMT. I suppose that the regular procedure is as the following. First, the coil orientation and location should be optimized to get the most prominent MEP at a TMS intensity higher than the RMT. Next, the RMT should be determined by some adaptive procedure reducing the TMS intensity. Finally, the intensity of TMS should be reduced to 90% of RMT, keeping the orientation and location of the coil. Is the authors' procedure different from this?

**Answer: **Yes, a very similar procedure to what the reviewer describes was used. First, the motor hotspot was defined as the cortical target producing the largest MEP amplitudes. Then, the motor threshold was determined as the intensity producing MEPs of 50 µV or larger 5 out of 10 times. Finally, the intensity was reduced to 90% of the RMT. However, if MEPs were still present, or the early TEP components were small (below 6 µV), or covered by muscle or decay artifacts, the coil was moved and/or rotated and the intensity adjusted to satisfy the following condition: [no MEPs] & [no artifacts after 15 ms] & [early TEP components bigger than 6 µV (before 50 ms)]. Details on the procedure can be found in Fecchio et al., 2017. We have clarified this procedure in the Data acquisition section: “Before the TMS–EEG experiment, we determined for each subject the optimal coil location and orientation producing the largest MEP with a fixed suprathreshold intensity [37], [38]. At the optimal location, we estimated the resting motor threshold (RMT) as the intensity producing MEPs larger than 50 µV in 5 out of 10 times [39].”

 How was the TMS intensity for pre-SMA optimized for each individual?

**Answer:** The TMS intensity was set to produce peak-to-peak amplitudes of 6–10 µV in the first 50 ms in the EEG signal. To achieve this, we used a graphical user interface (rt-TEP) to check in real-time for the quality of TEPs before starting the actual data acquisition (Casarotto et al., 2022). We utilized a functional mapping approach to set the stimulation parameters by delivering a few TMS pulse to check for the presence of TMS-evoked scalp muscle activations. If these were present, the stimulation parameters were adjusted to minimize the evoked muscle activity. Specifically, the coil was initially rotated, and if this procedure was not sufficient, the intensity was lowered. If also the last step was effective, we slightly translated the coil from the initial position. When TMS muscle activity was not detected, we proceeded delivering 20 pulses and checked for the presence of TEPs. The parameters of the stimulation were adjusted to obtain a signal with the first components lateralized (different from left to right), and with an amplitude in the channels under the stimulation site of 6–10 µV in the first 50 ms. This way, we could control both artefacts (described in more detail below and also in Casarotto et al., 2022)) and TEP amplitudes in real time. Please note that a similar procedure was followed for M1 targeting, although in this case we started from the hotspot location with the 90%MT and then, if the aforementioned conditions were not satisfied, we adjusted to new location and intensity as described a few lines above. We have clarified this information in the Data acquisition section:

 “The final stimulation parameters were adjusted based on the output of dedicated real-time EEG readout, a procedure followed as well for M1 (Casarotto et al., 2022).”

 I think that one of the challenges in TMS-EEG studies is artifact reduction. However, I don’t see details on the online and offline methodology of artifact reduction except for auditory artifact reduction using in-ear earphones and earmuffs. Were the lead wires rearranged relative to the coil orientation to reduce TMS-induced electromagnetic artifacts? How were the TMS-evoked artifacts, such as the decay artifact, removed in the offline analysis? Did the EEG amplifier system have any online artifact reduction protocol? Please explain these details more explicitly.

**Answer**: Most importantly, artifacts were avoided and reduced in the experimental session. The sample-and-hold amplifier cuts out the TMS pulse artifact, and there were no decay artifacts in the data. Muscle artifacts were avoided by careful coil placement, achieved by mapping as explained in the previous point. If present, decay artefacts were individuated by means of the rt-TEP tool and reduced/abolished by re-performing the impedances or rotating the position of the wires. Mapping was done by delivering 20 pulses and utilizing a graphical user interface (rt-TEP) to observe artifacts and peak-to-peak amplitudes (Casarotto et al., 2022). If the recorded TEPs were still affected by artifacts, the coil was rotated or moved to reduce them, and another 20 pulses were delivered. This was iterated until acceptable TEPs with minimal artifacts and large enough peak-to-peak amplitudes were achieved. Due to the careful methodology in the experiment, very clean data was recorded. Therefore, little needed to be removed in the offline analysis. We have added a clarifying sentence in the last paragraph of the Data acquisition section:

 “The sample-and-hold electronics of the EEG device [36], and the iterative process to adjust the coil location and orientation resulted in minimal TMS-related artifacts in the EEG recording for both stimulation locations.”

 On page 4, I understand that the authors conducted trial-wise permutation tests to analyze the GMFA differences between two target phase conditions within each subject. And the authors corrected the results by the number of multiple comparisons, which the authors think is 12, i.e., the number of datasets. However, if the authors conduct the permutation test at every time point, the number of comparisons will be much larger considering the number of time points. What is the rationale for ignoring the number of time points? Another idea is to go for the cluster-based permutation test (Maris *et al.* 2007 ^1^ ) considering the cluster. I am not sure if there is a single-subject version of the statistical test, though.

**Answer: **A separate multiple-comparison correction was utilized in the time domain than over the datasets. In the time domain, the correction was part of the trial-wise permutations, as the *maximum* differences between GMFA traces for each permutation was used to set the threshold for the whole time-window. Therefore, each sample is compared against the maximal statistic across time, thus controlling the family-wise error rate. This is a common method described, for example, by Holmes and colleagues (1996). We have now clarified this in the source analysis section: “This procedure controls the within-dataset false discovery rate (Holmes et al. 1996).”

 Figures 1 and 2. The titles for the figures look exactly the same. I guess the authors forgot to put M1 and pre-SMA.

**Answer: **Thank you for pointing this out. We have now specified the corresponding stimulation locations to each figure caption.

**References** Casarotto, S., Fecchio, M., Rosanova, M., Varone, G., D’Ambrosio, S., Sarasso, S., Pigorini, A., Russo, S., Comanducci, A., Ilmoniemi, R.J., Massimini, M., 2022. The rt-TEP tool: real-time visualization of TMS-Evoked Potentials to maximize cortical activation and minimize artifacts. J. Neurosci. Methods 370, 109486. https://doi.org/10.1016/j.jneumeth.2022.109486 Conforto, A.B., Z’Graggen, W.J., Kohl, A.S., Rösler, K.M., Kaelin-Lang, A., 2004. Impact of coil position and electrophysiological monitoring on determination of motor thresholds to transcranial magnetic stimulation. Clin. Neurophysiol. Off. J. Int. Fed. Clin. Neurophysiol. 115, 812–819. https://doi.org/10.1016/j.clinph.2003.11.010 Fecchio, M., Pigorini, A., Comanducci, A., Sarasso, S., Casarotto, S., Premoli, I., Derchi, C.-C., Mazza, A., Russo, S., Resta, F., Ferrarelli, F., Mariotti, M., Ziemann, U., Massimini, M., Rosanova, M., 2017. The spectral features of EEG responses to transcranial magnetic stimulation of the primary motor cortex depend on the amplitude of the motor evoked potentials. PloS One 12, e0184910. https://doi.org/10.1371/journal.pone.0184910 Holmes, A.P., Blair, R.C., Watson, J.D., Ford, I., 1996. Nonparametric analysis of statistic images from functional mapping experiments. J. Cereb. Blood Flow Metab. Off. J. Int. Soc. Cereb. Blood Flow Metab. 16, 7–22. https://doi.org/10.1097/00004647-199601000-00002 Reijonen, J., Pitkänen, M., Kallioniemi, E., Mohammadi, A., Ilmoniemi, R.J., Julkunen, P., 2020. Spatial extent of cortical motor hotspot in navigated transcranial magnetic stimulation. J. Neurosci. Methods 346, 108893. https://doi.org/10.1016/j.jneumeth.2020.108893 Wang, J., Meng, H.-J., Ji, G.-J., Jing, Y., Wang, H.-X., Deng, X.-P., Feng, Z.-J., Zhao, N., Zang, Y.-F., Zhang, J., 2020. Finger tapping task activation vs. TMS hotspot: different locations and networks. Brain Topogr. 33, 123–134. https://doi.org/10.1007/s10548-019-00741-9
